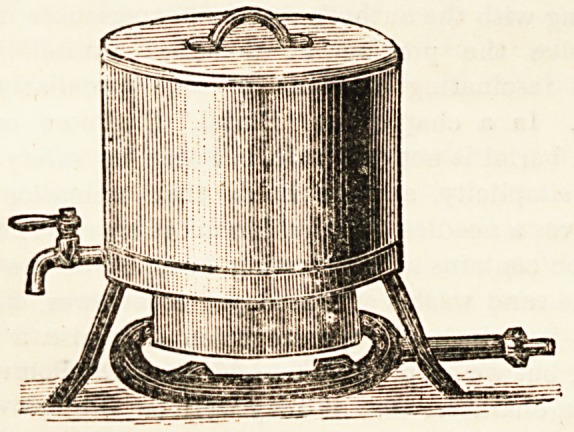# New Appliances and Things Medical

**Published:** 1903-10-10

**Authors:** 


### NEW APPLIANCES AND THINGS MEDICAL.

[We shall be glad to receive at our Office, 28 & 29 Southampton Street, Strand, London, W.C., from the manufacturers, specimens of all new preparations and appliances which may be brought out from time to time.]

### THE PYRO-KETTLE.

(The Boiler Heater Company, 46 Queen Victoria Street, London, E.C.)

This kettle differs greatly from the ordinary kettle found in household use. It is cylindrical in shape, with a flat lid, as shown in the illustration, and the tap is placed at its lowest zone, while inside a tinned plate partially attached divides the interior into two parts. When an ordinary kettle is heated the heat is communicated first to the lowest portion of the water, and spreads to the rest of the liquid principally by convection, so that the whole of the water must be made to boil before any water can be drawn off. With the pyro-kettle it is different, for the presence of the partition keeps the lower portion the hotter, and the water may here even be boiling while the upper portion of the liquid is only luke-warm. It is seen that the water in passing to the tap is exposed to the full source of heat, and cold water may be added slowly at the top without materially affecting the boiling water which is being drawn off; thus a continual supply can be kept up. It is therefore evident that the pyro-kettle should be of great use in hospitals, sick-rooms, etc., where hot water may be required at short notice, whilst it is also suitable for domestic use. Different sizes are made, either in tinned steel plate or copper—the price in both cases being most moderate.

### WHITE LABEL WHISKY.

(J. Dewar and Sons, Distillers, Perth. Dewar's Wharf, Waterloo Bridge, London, S.E.)

We are glad to have an opportunity of satisfying ourselves as to the wholesomeness of this whisky, for when so many deleterious brands are on the market it is of importance to the medical fraternity that they should know of a reliable one. This whisky is stated to be of great age and we have every reason to believe this statement to be true. Genuine old whisky is palatable and wholesome because those very products which give it a raw and disagreeable taste when young, are the very constituents which time, and time alone, fashions into pleasing flavour. There have been many attempts to artificially produce maturity by the removal of fusel oil, furfurol, and other aldehydes, but no success has ever been attained comparable with the mellowing influence of age, and any attempt to substitute such preparations is to be deplored and, on medical grounds, to be discountenanced strongly. There is naturally a great temptation to mature artificially, for long storage means so much capital locked up, besides some loss by evaporation. "White Label" whisky is of a pale sherry colour, indicating a small amount of extractives, while the degree of acidity is very slight. The flavour is delicate and mellow, with a complete absence of any harsh feeling to the palate, from which it may be deduced that it has been duly matured as stated. In medical practice, where alcoholic stimulation is deemed necessary, this whisky should certainly prove reliable and worthy of commendation.

## Figures and Tables

**Figure f1:**